# Effects of Using a Lumbar-Type Hybrid Assistive Limb by Older Adults With Frailty on Exercise Self-Efficacy: Retrospective Analysis Based on the Kanagawa Prospective ME-BYO Cohort Study

**DOI:** 10.2196/79358

**Published:** 2026-08-24

**Authors:** Makoto Nagasawa, Sho Nakamura, Yoshinobu Saito, Yuko Oguma, Takashi Kasukawa, Kaname Watanabe, Hiroto Narimatsu

**Affiliations:** 1 Cancer Prevention and Control Division, Kanagawa Cancer Center Research Institute, Yokohama, Kanagawa, Japan; 2 Graduate School of Health Innovation, Kanagawa University of Human Services, Research Gate Building Tonomachi 2-A, 3-25-10 Tonomachi, Kawasaki-ku, Kawasaki, Kanagawa, 210-0821, Japan, 81 44-589-8100; 3 Department of Genetic Medicine, Kanagawa Cancer Center, Yokohama, Kanagawa, Japan; 4 Faculty of Sport Management, Nippon Sport Science University, Kanagawa, Japan; 5 Graduate School of Physical Education, Health and Sport Studies, Nippon Sport Science University, Tokyo, Japan; 6 Center for Innovation Policy, Kanagawa University of Human Services, Kanagawa, Japan; 7 Sports Medicine Research Center, Keio University, Kanagawa, Japan; 8 Graduate School of Health Management, Keio University, Kanagawa, Japan; 9 Shonan Robocare Center, Kanagawa, Japan

**Keywords:** exercise self-efficacy, frailty, Hybrid Assistive Limb, lumbar type, physical activity, transtheoretical model

## Abstract

**Background:**

Improving exercise self-efficacy (ESE) is a key strategy for promoting sustained physical activity among older adults with frailty. The lumbar-type Hybrid Assistive Limb (HAL), a wearable device that supports voluntary movement through bioelectric signals, may facilitate mastery experiences in individuals with low baseline self-efficacy via adjustable assistance. However, its effects on ESE remain unclear.

**Objective:**

This study evaluated the effects of lumbar-type HAL exercise on ESE in older adults with frailty and examined whether responsiveness differed according to baseline readiness for exercise.

**Methods:**

A retrospective pooled analysis was conducted using data from 2 prospective studies of a lumbar-type HAL–based frailty prevention program: a feasibility study (n=20) and a randomized controlled trial (RCT; n=76; lumbar-type HAL group: n=38; waitlist control: n=38). Participants aged 50 to 84 years performed sit-to-stand and squat exercises using the lumbar-type HAL twice weekly. ESE was assessed using a validated 4-item questionnaire (range 4‐20). The prespecified primary analysis examined within-person changes in ESE in the lumbar-type HAL group (2-tailed paired *t* test; n=96). A secondary analysis compared changes in ESE between the lumbar-type HAL and waitlist control groups in the RCT using the Welch *t* test and baseline-adjusted analysis of covariance. A prespecified subgroup analysis compared participants in stages 1 to 4 of the transtheoretical model of behavior change (exercise habit not established) with those in stage 5 (maintenance). All statistical tests were 2 sided, with significance at *P*<.05.

**Results:**

The primary pooled paired analysis demonstrated a significant increase in ESE of 1.05 points (95% CI 0.41-1.69; *P*=.002). The subgroup analysis was also significant: those in stages 1 to 4 (n=56) improved by 1.73 points vs 0.05 points for those in stage 5 (n=38), a difference of 1.68 points (95% CI 0.41-2.95; *P*=.01). The largest within-group improvement was observed in stage 1 (precontemplation: +3.50 points, 95% CI 0.27-6.73; *P*=.04; Cohen *d*=1.14); 4 of 6 participants improved and none deteriorated. In the secondary RCT between-group comparison, the lumbar-type HAL group showed a directionally favorable improvement (between-group difference=1.21 points, 95% CI −0.33 to 2.75; *P*=.12; Cohen *d*=0.36). The subgroup differences were 2.10 points in stages 1 to 4 (*P*=.07; Cohen *d*=0.58) and 0.02 points in stage 5 (*P*=.99). Sensitivity analyses confirmed robustness to source-related heterogeneity (heterogeneity test: *P*=.74; source-adjusted regression: *P*=.76).

**Conclusions:**

The pooled analysis demonstrated significant within-person improvement in ESE among older adults with frailty following lumbar-type HAL training, with the greatest improvement in individuals without established exercise habits. The RCT indicated a favorable but inconclusive between-group effect, and adequately powered confirmatory RCTs remain necessary. The exploratory response among precontemplators—typically difficult to engage in interventions—suggests that HAL-supported mastery experiences may support disengaged older adults at high risk of frailty in starting physical exercise.

## Introduction

### Frailty and Exercise Self-Efficacy in Older Adults

Frailty is a common clinical syndrome in older adults and is associated with increased risk of hospitalization, falls, and disability, highlighting the need for targeted interventions [1,2]. Exercise self-efficacy (ESE), a psychological construct linked to frailty [3], reflects confidence in maintaining exercise despite barriers [4]. Lower ESE is associated with reduced physical activity (PA) [3], supporting its relevance as an outcome in exercise interventions. According to the social cognitive theory by Bandura [5], self-efficacy is shaped by mastery experiences, vicarious experiences, verbal persuasion, and physiological and affective states, with mastery experiences being most influential [5,6]. Mastery experiences involve successful performance in challenging tasks. In rehabilitation, task difficulty is adjusted to enable success, supporting motor learning and improving efficiency [7]. Thus, appropriate task adjustment may facilitate mastery experiences and enhance ESE.

### Challenge of Reaching Disengaged Older Adults

Behavioral readiness for exercise, as defined by the transtheoretical model (TTM) of behavior change, is a key determinant of intervention responsiveness [8]. Older adults in precontemplation (stage 1)—who have no intention of initiating exercise—are typically difficult to engage in conventional exercise programs and rarely accumulate mastery experiences. Although exercise interventions can improve physical function and frailty status in older adults [9], systematic reviews of TTM-based interventions report limited and inconsistent evidence for promoting PA in this population [10]. Therefore, how intervention responsiveness varies by baseline behavioral readiness remains poorly understood.

### Lumbar-Type Hybrid Assistive Limb and Mastery Experience

The Hybrid Assistive Limb (HAL; Cyberdyne Inc) is a wearable cyborg that supports movement in response to voluntary intent [11,12]. It uses skin-mounted sensors to detect bioelectric signals associated with movement intention and converts these signals into motor torque assistance. Unlike conventional exoskeletons that rely primarily on preprogrammed motion patterns, the HAL generates assistance directly in response to the wearer’s voluntary activity [13]. Clinical studies have shown that a lower limb–type HAL improves walking ability in patients with stroke and spinal cord injury [13-17]. The lumbar-type HAL consists of an exoskeleton frame and a power unit comprising angle sensors and actuators for both hip joints. It assists hip extension during transitions from flexion to extension, supporting standing and lifting movements. Compared with the lower limb type, it is lighter and more compact, enabling its use in settings such as home-based training and group exercise programs in long-term care. Previous studies in frail populations have reported improvements in walking speed and stride length [18], although evidence in older adults with frailty remains limited. By providing consistent, scaffolded assistance, the lumbar-type HAL may facilitate mastery experiences—particularly in individuals with low baseline self-efficacy—thereby enhancing ESE. However, its effect on ESE has not yet been examined.

### Study Aim and Hypotheses

This study aimed to evaluate the effects of a lumbar-type HAL exercise program on ESE in older adults with frailty: (1) lumbar-type HAL training was hypothesized to improve ESE in this population, and (2) the magnitude of improvement was hypothesized to vary by baseline TTM stage, with the greatest gains expected among participants who had not yet established a regular exercise habit (stages 1‐4) compared with those in the maintenance stage (stage 5).

## Methods

### Study Design and Data Source

**Figure 1.**
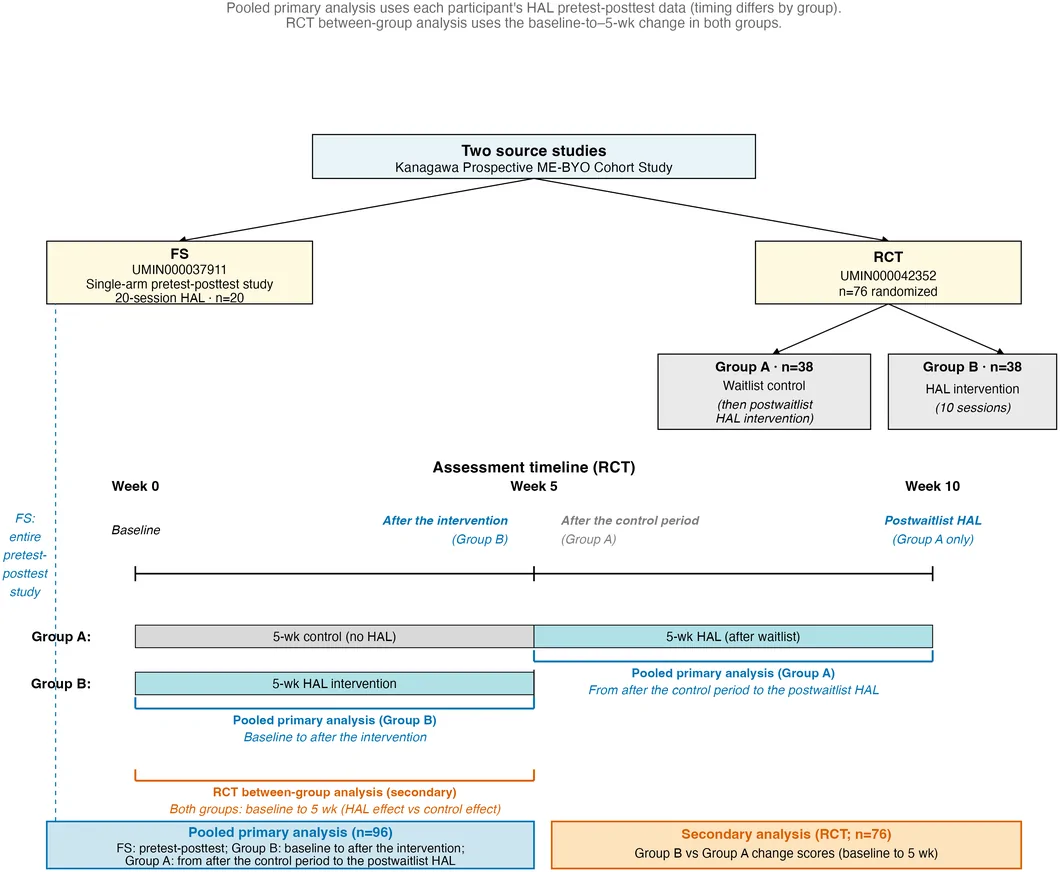
Participant flow and analytic use of assessment time points. FS: feasibility study; HAL: Hybrid Assistive Limb; RCT: randomized controlled trial.

This study used a retrospective secondary analysis of pooled outcome data derived from 2 prospective studies of a lumbar-type HAL–based care prevention program (see Figure 1 for the participant flow). The source studies were a feasibility study (FS; UMIN000037911) and a randomized controlled trial (RCT; UMIN000042352) based on the Kanagawa Prospective ME-BYO Cohort Study entitled “Research on the development of a nursing care prevention program using healthcare robots” [19]. Data from both the FS and the RCT were pooled to evaluate the effect of a lumbar-type HAL exercise program on ESE.

The FS aimed to evaluate the feasibility and effectiveness of a care prevention program using the lumbar-type HAL. The primary end point comprised program completion rate. Participants aged below 85 years who met at least one of the frailty criteria based on the Japanese version of the Cardiovascular Health Study, including weight loss, fatigue, reduced PA level, decreased grip strength, and slow walking speed, were included in the analysis. The intervention consisted of lumbar-type HAL training delivered twice weekly for 90 minutes per session over 20 sessions. Ultimately, 20 participants completed the program.

The RCT evaluated the effectiveness of lumbar-type HAL training for care prevention, with normal walking speed as the primary end point. A randomized, single-blind, parallel-group design was used, with participants randomly assigned to either the intervention group or a waitlist control group. Individuals aged 65 to 85 years with a score of 6 or higher on the 5-question Geriatric Locomotive Function Scale, a normal walking speed of less than 1.0 m/s (eligibility based on either the Geriatric Locomotive Function Scale or walking speed criteria), and no certification for long-term care were analyzed. The intervention group received lumbar-type HAL training twice weekly for 90 minutes per session over 10 sessions, whereas the waitlist control group received no intervention during the 5-week period [19]. Ultimately, 77 participants completed the study.

### Intervention: Exercise Program Using Lumbar-Type HAL

In both the FS and the RCT, lumbar-type HAL training was conducted primarily in cybernic voluntary control mode, in which assistive torque supporting hip extension during standing and lifting tasks was generated in response to bioelectric signals detected by skin surface sensors placed over the erector spinae muscles. Assistance levels were individually adjusted according to each participant’s functional capability to ensure appropriate support during training. The program included repetitive exercises such as sit-to-stand movements and squats.

### Assessment Measures

Assessment measures included ESE, the 10-m walk test (10MWT), the timed up and go (TUG) test, amount of PA, TTM stage of behavior change [8], and a self-administered questionnaire. ESE was assessed using a 4-item questionnaire developed by Oka [4] evaluating confidence in exercising when slightly tired, unmotivated, busy, or under unfavorable weather conditions. Responses were rated on a 5-point Likert scale (1=not at all confident; 5=very confident), with higher scores indicating greater ESE. In the 10MWT, participants walked at a usual pace along a 10-m walkway, and walking speed (m/s) was calculated using stopwatch timing. The TUG test measured the time required to rise from a chair, walk 3 m, turn, return, and sit. PA was assessed using 2 items: weekly exercise frequency (5‐7 days, 3‐4 days, 1‐2 days, or none) and daily exercise duration. Average daily duration was estimated by multiplying frequency (coded as 6, 3.5, 1.5, and 0 days per week) by duration per day and dividing by 7 [20]. TTM stage was rated on a 5-point scale: stage 1 (precontemplation: no intention to perform PA), stage 2 (contemplation: planning to start soon), stage 3 (preparation: performing activity once a week), stage 4 (action: regular activity for <6 months), and stage 5 (maintenance: regular activity for ≥6 months). Regular PA was defined as engaging in PA for 60 minutes per day or more (≥40 minutes for individuals aged ≥65 years) even at low intensity. The self-administered questionnaire was structured as an interview to capture participants’ subjective perceptions of changes after program completion. Data from both studies were pooled for analysis, with ESE as the primary end point and the 10MWT, the TUG test, PA, and questionnaire responses as secondary end points.

### Sample Size Calculation

Sample size calculation was based on ESE data from the FS (mean change 1.25, SD 2.88 points; paired Cohen *d*=0.43) [21]. For the primary pooled within-person analysis, 44 participants were required to achieve 80% power with a 2-sided α of .05; the achieved sample size (n=96) exceeded this requirement. For the secondary RCT between-group comparison, 172 participants (86 per group) were required; the achieved sample size (n=76) was below this threshold, resulting in limited statistical power (post hoc: 30%‐34% at a 2-sided α=.05).

### Statistical Analysis

#### Overview

Descriptive data were expressed as means and SDs for continuous variables and numbers and percentages for categorical variables. The 95% CIs were reported for all primary effect estimates. All statistical tests were 2 sided, with a significance threshold of .05. Statistical analyses were performed using the R software (version 4.2.2; R Foundation for Statistical Computing). One participant with incomplete primary outcome data was excluded from the analysis.

#### Primary Analysis

The primary analysis comprised a within-person pretest-posttest comparison of ESE among all lumbar-type HAL recipients (n=96) pooled from the FS and the RCT (the RCT intervention group plus the waitlist control group after completion of the lumbar-type HAL exercise program) using a paired *t* test. In the RCT, ESE was assessed at baseline (week 0), after the intervention (week 5), and—for the waitlist control group only—after the subsequent 5-week lumbar-type HAL program (postwaitlist HAL; week 10). For the pooled primary analysis, each participant contributed a pretest-posttest comparison corresponding to lumbar-type HAL exposure: from baseline to week 5 for the intervention group (group B) and from after the control period to after the intervention for the waitlist group (group A; Figure 1). The waitlist group received lumbar-type HAL training after the control period for ethical reasons in accordance with the registered protocol (UMIN000042352) [19]. This pooled pretest-posttest comparison constituted the primary inferential analysis specified in the study design, which was powered to detect changes in ESE, an outcome not individually powered in either source study. Paired *t* tests were also applied to the 10MWT, TUG, and PA outcomes.

#### Subgroup Analyses

An a priori dichotomous subgroup comparison was specified prior to pooled data analysis based on TTM theory [8] and findings from the FS. The comparison contrasted stages 1 to 4 (precontemplation, contemplation, preparation, and action; no established exercise habit) vs stage 5 (maintenance; regular exercise for ≥6 months). This contrast was not included in the original University Hospital Medical Information Network Clinical Trials Registry protocols (UMIN000037911 and UMIN000042352) as those preceded the development of the pooled analysis plan; however, specification occurred prior to data integration from the 2 source studies. The empirical rationale was derived from the FS, in which participants in lower-readiness stages (n=10) demonstrated a mean ESE increase of 2.5 points compared with 0 points in higher-readiness participants (n=10; *P*=.052) [21]. The theoretical basis aligns with the social cognitive theory by Bandura [5], which predicts stronger mastery-related effects among individuals with lower baseline self-efficacy, and with the TTM framework, in which stage 5 reflects a maintenance phase associated with attenuated intervention effects due to ceiling effects. Within-stratum changes in the pooled dataset were analyzed using paired *t* tests, and between-stratum differences in change scores were assessed using the Welch *t* test. ESE changes were also described across all 5 TTM stages; given the small sample sizes within some stages, these analyses were considered exploratory.

#### Secondary Analysis (RCT; Between Groups)

The randomized design of the RCT (n=76) was used as a secondary analysis to compare ESE change over the same 5-week period between the lumbar-type HAL group (n=38) and the waitlist control group (n=38). Between-group differences in change scores were assessed using the Welch *t* test. Results were further confirmed using analysis of covariance, with postintervention ESE as the dependent variable, group as the fixed factor, and baseline ESE as a covariate. The Cohen *d* was calculated as a standardized effect size. The prespecified TTM dichotomy was also examined within this between-group framework.

#### Sensitivity Analyses for the Pooled Estimate

Robustness of the pooled estimate was evaluated against potential heterogeneity between the source studies (FS: 20 sessions, single arm; RCT: 10 sessions with a waitlist control group) using three prespecified sensitivity analyses within a fixed-effects framework: (1) source-stratified paired *t* tests performed separately within each study, (2) source-adjusted linear regression including a binary study indicator (FS=1; RCT=0), and (3) a between-study heterogeneity assessment using the Welch *t* test on change scores comparing FS and RCT participants.

### Ethical Considerations

Both source studies were approved by the Research Ethics Review Committee of the Graduate School of Health Innovation, Kanagawa University of Human Services (FS: approval number 20019-36-004; RCT: approval number Hodai 30-008). Written informed consent was obtained from all participants, including permission for secondary analyses of deidentified data. The present pooled analysis was reviewed by the same committee and deemed exempt from full ethics review (review notification number SHI 62) as it used existing deidentified data. This study was conducted in accordance with the Declaration of Helsinki.

## Results

### Participant Characteristics

ESE data were available for 99% (96/97) of the participants who completed either the FS or RCT programs. Participants’ age ranged from 50 to 84 years. Baseline characteristics of these participants are shown in Table 1.

**Table 1.** Participant characteristics (n=96)^a^.

Characteristics	Participants, n (%)
Age (y)	
50‐59	4 (4.2)
60‐69	10 (10.4)
70‐79	66 (68.8)
80‐84	16 (16.7)
Sex (n=95)	
Male	28 (29.5)
Female	67 (70.5)
Source study	
FS^b^	20 (20.8)
RCT^c^	76 (79.2)
Stage of TTM^d^ (n=94)	
Stage 1 (precontemplation)	6 (6.4)
Stage 2 (contemplation)	28 (29.8)
Stage 3 (preparation)	13 (13.8)
Stage 4 (action)	9 (9.6)
Stage 5 (maintenance)	38 (40.4)

a Sex was not available for 1% (1/96) of the participants. Transtheoretical model stage data were missing for 2.1% (2/96) of the participants (1 in the postwaitlist group A and 1 in the randomized controlled trial lumbar-type Hybrid Assistive Limb group).

b FS: feasibility study.

c RCT: randomized controlled trial.

d TTM: transtheoretical model.

### Pooled Pretest-Posttest Analysis (Primary Analysis)

In the primary pooled within-person analysis of all lumbar-type HAL recipients (n=96), ESE increased significantly by 1.05 points (95% CI 0.41-1.69; *P*=.002) from a baseline mean of 11.94 (SD 3.05) points to 12.99 (SD 3.21) points after the intervention (Table 2). Secondary outcomes also showed significant improvement: 10MWT walking speed increased by 0.37 m/s (95% CI 0.33-0.40; *P*<.001), TUG time decreased by 1.68 seconds (95% CI −2.01 to −1.35; *P*<.001), and PA increased by 18.28 minutes per day (95% CI 12.95-23.62; *P*<.001; Table 2).

**Table 2.** Effect of lumbar-type Hybrid Assistive Limb training on primary and secondary outcomes (pooled paired analysis; n=96)^a^.

Outcome	Participants, n	Baseline, mean (SD)	After the intervention, mean (SD)	Change, mean (95% CI)	*P* value	Cohen *d*
ESE^b^ total score (range 4‐20)	96	11.94 (3.05)	12.99 (3.21)	+1.05 (+0.41 to +1.69)	.002	0.33
10MWT^c^ speed (m/s)	96	0.99 (0.28)	1.36 (0.37)	+0.37 (+0.33 to +0.40)	<.001	—^d^
TUG^e^ time (s)	96	10.12 (8.47)	8.45 (7.64)	–1.68 (–2.01 to –1.35)	<.001	—
PA^f^ (min per d)	96	30.47 (29.27)	48.75 (33.99)	+18.28 (+12.95 to +23.62)	<.001	—

a Exercise self-efficacy is the primary outcome; 10-m walk test speed, timed up and go time, and physical activity are secondary outcomes. Pooled paired analysis (n=96): paired *t* test, 2-sided α=.05. The 10-m walk test speed (m/s) was calculated as 10 m divided by walking time.

b ESE: exercise self-efficacy.

c 10MWT: 10-m walk test.

d Cohen *d* is reported only for the primary outcome (ESE).

e TUG: timed up and go test.

f PA: physical activity.

Sensitivity analyses supported the robustness of the pooled estimate to source-related heterogeneity (Table 3). Source-stratified analyses demonstrated similar improvements (FS: Δ=+1.25 points, *P*=.07, and n=20; RCT lumbar-type HAL group: Δ=+1.00 point, *P*=.009, and n=76). The between-source heterogeneity test was nonsignificant (*P*=.74), and source-adjusted regression produced a comparable estimate (+1.00 point) with a nonsignificant source effect (*P*=.76).

**Table 3.** Sensitivity analyses for the pooled estimate of exercise self-efficacy change^a^.

Analysis	Participants, n	Point estimate (95% CI)	*P* value
Primary pooled paired analysis	96	+1.05 (+0.41 to +1.69)	.002
Source stratified			
FS^b^ only	20	+1.25 (−0.10 to +2.60)	.07
RCT^c^ lumbar-type HAL^d^ recipients	76	+1.00 (+0.26 to +1.74)	.009
Between-source heterogeneity^e^			
FS − RCT difference	96	+0.25 (−1.26 to +1.76)	.74
Source-adjusted regression^f^			
Pooled estimate (RCT reference)	96	+1.00 (+0.28 to +1.72)	.007
Source coefficient (FS vs RCT)	—^g^	+0.25 (−1.33 to +1.83)	.76

a Prespecified sensitivity analyses for the pooled primary estimate. A fixed-effects framework was applied as the 2 source studies represent sequential phases of a single research program*.*

b FS: feasibility study.

c RCT: randomized controlled trial.

d HAL: Hybrid Assistive Limb.

e Between-source heterogeneity test: Welch *t* test comparing change scores between FS and RCT participants.

f Source-adjusted regression: linear regression of exercise self-efficacy change on a source-study indicator (FS=1; RCT=0)*. *The intercept estimate represents the pooled mean change, with RCT as the reference. The source coefficient tests for between-source difference.

g Not applicable (the source coefficient tests the between-source difference within the regression model including all 96 participants and does not correspond to a separate participant subgroup).

### Pooled TTM Subgroup Analysis

Subgroup analyses stratified by baseline TTM stage were conducted to characterize changes in ESE (Table 4). Participants in the precontemplation stage showed a mean increase of 3.50 (SD 3.08) points following lumbar-type HAL training. The mean ESE increases were 1.14 (SD 3.27), 1.46 (SD 2.79), and 2.78 (SD 3.56) points in the contemplation (n=28), preparation (n=13), and action (n=9) stages, respectively. In contrast, the maintenance stage (n=38) showed a minimal change of 0.05 (SD 2.86) points. The prespecified between-stratum comparison was significant. Stages 1 to 4 combined (n=56) demonstrated a mean ESE increase of 1.73 points (95% CI 0.87-2.60; *P*<.001) compared with 0.05 points in stage 5 (n=38; 95% CI −0.90 to 1.01; *P*=.91), yielding a between-stratum difference of +1.68 points (95% CI 0.41-2.95; *P*=.01; Table 4).

**Table 4.** Change in exercise self-efficacy by baseline transtheoretical model stage (pooled analysis; n=94)^a^.

	Participants, n	Baseline score (4-20), mean (SD)	Postintervention score (4-20), mean (SD)	Change (points), mean (95% CI)	*P* value
Stage 1 (precontemplation)	6	8.00 (3.16)	11.50 (4.18)	+3.50 (+0.27 to +6.73)	.04
Stage 2 (contemplation)	28	11.46 (3.17)	12.61 (3.65)	+1.14 (−0.13 to +2.41)	.08
Stage 3 (preparation)	13	12.00 (2.58)	13.46 (2.67)	+1.46 (−0.22 to +3.15)	.08
Stage 4 (action)	9	12.22 (2.28)	15.00 (3.32)	+2.78 (+0.04 to +5.52)	.047
Stage 5 (maintenance)	38	12.76 (2.89)	12.82 (2.81)	+0.05 (−0.90 to +1.01)	.91
Stage 1‐4 (combined)	56	11.34 (3.08)	13.07 (3.51)	+1.73 (+0.87 to +2.60)	<.001
Difference (stages 1‐4 vs 5)	—^b^	—	—	+1.68 (+0.41 to +2.95)	.01

a Paired *t *tests within each subgroup; between-stratum comparisons were performed using the Welch *t *test on change scores. Two-sided α=.05. Per-stage analyses are exploratory and hypothesis generating due to the small sample sizes within several stages.

b Baseline and postintervention values are not applicable for the between-stratum difference row as it reflects a contrast between groups rather than a within-group estimate.

### RCT Between-Group Comparison (Secondary Analysis)

**Figure 2.**
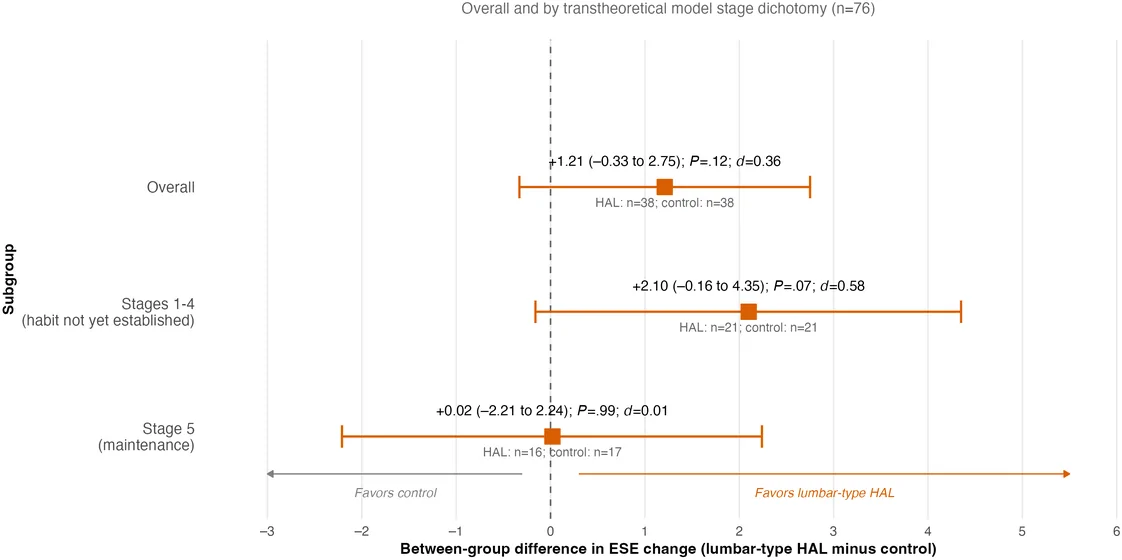
Randomized controlled trial between-group comparison of exercise self-efficacy (ESE) change. HAL: Hybrid Assistive Limb.

The secondary analysis used the randomized design of the RCT to support causal inference (Table 5 and Figure 2). The lumbar-type HAL group (n=38) showed a mean ESE change of +1.16 (SD 3.67) points compared with −0.05 (SD 3.05) points in the waitlist control group (n=38). The between-group difference in change was 1.21 points (95% CI −0.33 to 2.75; *P*=.12; Cohen *d*=0.36). Analysis of covariance adjusting for baseline ESE yielded a similar adjusted between-group difference of 1.08 points (95% CI −0.28 to 2.44; *P*=.12). Limited statistical power was present for this between-group comparison (post hoc: 30%‐34% at α=.05), and interpretation relied on consistency with the pooled primary estimate. Within the prespecified TTM dichotomy, the between-group difference was 2.10 points among participants in stages 1 to 4 (lumbar-type HAL: n=21; control: n=21; 95% CI −0.16 to 4.35; *P*=.07; Cohen *d*=0.58) and 0.02 points among participants in stage 5 (lumbar-type HAL: n=16; control: n=17; 95% CI −2.21 to 2.24; *P*=.99; Cohen *d*=0.01; Table 5 and Figure 2). Stage-specific descriptive results are provided in Multimedia Appendix 1.

**Table 5.** Effect of lumbar-type Hybrid Assistive Limb (HAL) training on exercise self-efficacy in the randomized controlled trial: between-group comparison overall and by transtheoretical model stage dichotomy^a^.

Group and subgroup	Participants, n	Baseline score (4-20), mean (SD)	Postintervention score (4-20), mean (SD)	Change (points), mean (SD)	Difference (95% CI)	*P* value	Cohen *d*
Overall					+1.21 (−0.33 to +2.75)	.12	0.36
Lumbar-type HAL	38	11.63 (2.84)	12.79 (3.31)	1.16 (3.67)			
Waitlist control	38	11.87 (3.19)	11.82 (3.21)	−0.05 (3.05)			
Stage 1-4^b^					+2.10 (−0.16 to +4.35)	.07	0.58
Lumbar-type HAL	21	10.57 (2.64)	12.33 (3.65)	1.76 (3.81)			
Waitlist control	21	11.67 (3.15)	11.33 (3.06)	−0.33 (3.41)			
Stage 5^c^					+0.02 (−2.21 to +2.24)	.99	0.01
Lumbar-type HAL	16	12.88 (2.66)	13.19 (2.86)	0.31 (3.53)			
Waitlist control	17	12.12 (3.31)	12.41 (3.39)	0.29 (2.59)			

a Between-group difference (lumbar-type HAL minus waitlist control) was calculated using the Welch *t *test on change scores. Analysis of covariance adjusting for baseline exercise self-efficacy yielded similar results: +1.08 points (95% CI −0.28 to +2.44; *P*=.12)*.*

b Stages 1-4: precontemplation, contemplation, preparation, and action stages (regular exercise habit not yet established).

c Stage 5: maintenance stage (regular exercise habit established for ≥6 months).

### Subjective Feedback

Responses to the self-administered questionnaire included individual statements such as the following:

My motivation to exercise has increased.

I want to continue exercising in the future.

It has become easier to move. Standing up takes less effort now.

I can stand up without having to hold on.

I felt that my back was more comfortable after using the HAL for the first time. My sitting posture has improved.

## Discussion

### Principal Findings

This retrospective pooled analysis combining an FS and an RCT of lumbar-type HAL training in older adults with frailty demonstrated a significant improvement in ESE of 1.05 points (95% CI 0.41-1.69; *P*=.002) among 96 recipients with lumbar-type HAL. Improvements in ESE were accompanied by significant gains in physical function (10MWT and TUG test) and PA, indicating concurrent enhancement of physical and psychological readiness for exercise. Improvements in physical function likely reinforced mastery experiences, thereby contributing to increased ESE [5-7]. By providing consistent, scaffolded assistance, the lumbar-type HAL enables successful completion of functional tasks such as sit-to-stand movements and squats that may otherwise exceed baseline capacity, thereby strengthening confidence in performing exercise under everyday conditions.

**Figure 3.**
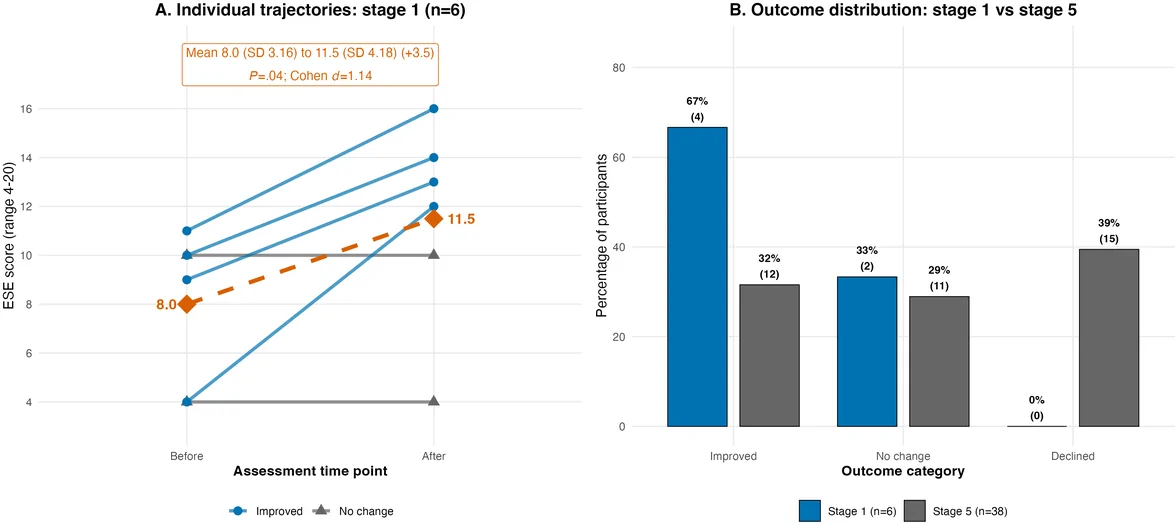
Stage 1 (precontemplation) detailed analysis. ESE: exercise self-efficacy.

Subgroup analyses demonstrated differential effects across baseline TTM stages. The prespecified between-stratum contrast (stages 1‐4 vs stage 5) was significant (between-stratum difference of +1.68 points, 95% CI 0.41-2.95; *P*=.01), indicating that responsiveness varied by behavioral readiness. The largest effect was observed in precontemplation (stage 1), where 4 of 6 participants improved (none deteriorated; mean change +3.50, SD 3.08 points; Cohen *d*=1.14; Figure 3). This exploratory finding (n=6) is of interest given that precontemplators are typically difficult to engage in conventional exercise programs and have limited opportunities for mastery experiences [6,10]. In contrast, participants in stage 5 (maintenance) showed minimal change (+0.05 points; *P*=.91). Although baseline ESE in this subgroup did not approach the scale maximum, it was already relatively high for this population, leaving little room for further improvement; in addition, the intervention tasks (sit-to-stand exercises and squats) may have provided limited additional challenge for individuals with established exercise habits [22]. Sensitivity analyses supported the validity of pooling FS and RCT data (between-source heterogeneity: *P*=.74; source-adjusted regression: *P*=.76; RCT-only results were directionally consistent), suggesting minimal influence of methodological differences between studies. The secondary RCT between-group analysis was not significant (between-group difference of +1.21 points; *P*=.12; Cohen *d*=0.36), likely reflecting limited statistical power (post hoc: 30%‐34%; required n=172 for 80% power). However, the effect estimate aligned closely with the pooled primary result (+1.21 vs +1.05 points), and the TTM pattern was consistent (Cohen *d*=0.58 for stages 1‐4 vs Cohen *d*=0.01 for stage 5); thus, the direction of the effect estimates was consistent across analyses rather than contradictory. However, confirmation through a sufficiently powered, dedicated RCT is required before causal inference can be established.

### Comparison With Previous Studies

These findings extend those of previous studies on the lumbar-type HAL by demonstrating effects on a psychological outcome (ESE) in addition to previously reported physical function [18]. To our knowledge, this study is the first to examine lumbar-type HAL responsiveness according to TTM stage and describe an exploratory finding among precontemplators. The observed effect size in this subgroup (Cohen *d*=1.14) aligns with the theory by Bandura [5] that mastery experience interventions yield the largest gains among individuals with low baseline self-efficacy. The minimal change observed in the maintenance stage is consistent with prior exercise intervention studies in older adults with established activity habits, in which ceiling effects on self-efficacy measures are commonly reported [22]. A recent systematic review reported limited and inconsistent evidence on TTM-based interventions for PA in older adults [10], highlighting the scarcity of evidence on stage-tailored exercise strategies. The present findings provide preliminary evidence that lumbar-type HAL training may serve as a stage-appropriate entry strategy for previously disengaged individuals. Notably, a recent study of a wearable walking assistance robot in community-dwelling older adults [23] reported feasibility and acceptability without examining ESE; the present study extends this line of research by linking robot-assisted exercise to a psychological readiness outcome.

### Limitations

This study has some limitations. First, the FS and RCT differed in the structure and delivery of the lumbar-type HAL–based exercise programs. The FS implemented an individualized program, whereas the RCT combined individual and group-based training. Group-based settings provide opportunities for social encouragement and observational learning (vicarious experiences), both of which enhance self-efficacy [5]. Accordingly, participants in the RCT may have had greater exposure to self-efficacy–enhancing mechanisms than those in the FS. Moreover, the number of lumbar-type HAL training sessions differed between studies (20 in the FS vs 10 in the RCT), which may have influenced the magnitude of ESE improvement. These heterogeneities were addressed through prespecified sensitivity analyses (source stratified, source-adjusted regression, and between-source heterogeneity testing), all of which supported the robustness of the pooled estimate. Second, the secondary RCT between-group analysis was underpowered: the achieved sample size (n=76; 38 per group) was below the required 172 to detect a medium effect (Cohen *d*=0.43) at a 2-sided α of .05 with 80% power, corresponding to a post hoc power of 30% to 34%. The directionally consistent effects observed in the pooled primary analysis (significant; +1.05 points; *P*=.002) and the secondary RCT analysis (nonsignificant; +1.21 points; *P*=.12) suggest a potentially meaningful intervention effect; however, a larger adequately powered RCT is required for definitive between-group inference. Third, subgroup analyses by TTM stage were limited by small sample sizes within some strata (eg, stage 1: n=6 in the pooled analysis; n=2 in the lumbar-type HAL group and n=4 in the control group of the RCT). Consequently, stage-specific findings should be considered exploratory and hypothesis generating. The prespecified dichotomous comparison between stages 1 to 4 vs stage 5 (*P*=.01) therefore served as the primary stratified analysis. Although no participant in stage 1 showed a decline in ESE, confirmation in studies adequately powered to assess effects within individual TTM stages remains necessary. Fourth, open recruitment may have preferentially attracted participants with greater health awareness, potentially limiting the generalizability of the findings.

### Future Directions

Future research should recruit larger numbers of participants in the precontemplation stage (stage 1) to confirm the large effect estimate observed in this exploratory analysis (n=6; Cohen *d*=1.14). A purposive sampling strategy targeting disengaged older adults—who are often underrepresented in exercise research—would enable rigorous evaluation of whether the pronounced ESE gains observed in precontemplators can be replicated. Adequately powered studies stratified by baseline TTM stage are needed to determine whether lumbar-type HAL training reliably benefits older adults with low readiness for exercise. The differential effects observed across TTM stages also support a stratified intervention approach whereby lumbar-type HAL training may be appropriately targeted to lower-readiness individuals (stages 1‐4), whereas alternative strategies may better serve those in the maintenance stage.

### Conclusions

In this retrospective pooled analysis, a lumbar-type HAL exercise program demonstrated a significant within-person improvement in ESE in older adults with frailty, with the greatest improvement observed among individuals without established exercise habits. Evidence from the RCT indicated a favorable but inconclusive between-group effect, and adequately powered confirmatory RCTs remain necessary.

The exploratory response in precontemplators—a group traditionally difficult to engage in exercise interventions—suggests that HAL-supported mastery experiences may provide a novel entry point to PA for disengaged older adults at high risk of frailty. Future studies should specifically target participants in stage 1 using purposive sampling, confirm the differential effects in adequately powered stratified designs, and examine whether improvements in ESE translate into sustained PA over time.

## Supplementary material

10.2196/79358 Multimedia Appendix 1 Stage-by-stage descriptive results from the randomized controlled trial stratified by transtheoretical model stage (1-5) and intervention group.

## Abbreviations

10MWT: 10-m walk test
ESE: exercise self-efficacy
FS: feasibility study
HAL: Hybrid Assistive Limb
PA: physical activity
RCT: randomized controlled trial
TTM: transtheoretical model
TUG: timed up and go
