# Supplementary material for: Effects of Using a Lumbar-Type Hybrid Assistive Limb by Older Adults With Frailty on Exercise Self-Efficacy: Retrospective Analysis Based on the Kanagawa Prospective ME-BYO Cohort Study
Source: JMIR Rehabil Assist Technol. 2026 Aug 24;13:e79358. doi: 10.2196/79358 (PMC13507523; doi:10.2196/79358)
Supplement: Supplementary file 1 [file rehab-v13-e79358-s001.docx]

## Multimedia Appendix 1. Stage-by-stage descriptive results from the randomized controlled trial, stratified by transtheoretical model stage (1–5) and intervention group

|  | **lumbar-type HAL** | | **Waitlist control** | |  |  |  |
| --- | --- | --- | --- | --- | --- | --- | --- |
| **TTM stage** | **n** | **Change (SD)** | **n** | **Change (SD)** | **Between-group difference** | **95% CI** | ***P*** |
| Stage 1 (precontemplation) | 2 | +2.50 (3.54) | 4 | −1.25 (2.75) | +3.75 | −11.42 to +18.92 | *P*=.34 |
| Stage 2 (contemplation) | 11 | +1.45 (3.62) | 5 | −1.00 (3.16) | +2.45 | −1.59 to +6.50 | *P*=.20 |
| Stage 3 (preparation) | 5 | +2.00 (4.18) | 8 | +1.62 (3.66) | +0.38 | −4.90 to +5.65 | *P*=.87 |
| Stage 4 (action) | 3 | +2.00 (6.08) | 4 | −2.50 (2.65) | +4.50 | −8.65 to +17.65 | *P*=.33 |
| Stage 5 (maintenance) | 16 | +0.31 (3.53) | 17 | +0.29 (2.59) | +0.02 | −2.21 to +2.24 | *P*=.99 |
| *Difference: lumbar-type HAL change minus waitlist control change.* | | | | | | | |
| *Welch t test on change scores. The individual per-stage between-group differences for stages 1–4 should be interpreted with caution, as at least one group in each of these stages had a small cell size (n<10); the correspondingly wide confidence intervals reflect this limited precision. These per-stage results are descriptive and exploratory and complement, but do not replace, the prespecified between-stratum contrast comparing the combined stages 1–4 with stage 5 (P=.01) reported in the main text.* | | | | | | | |
| *ESE: exercise self-efficacy; TTM: transtheoretical model; CI: confidence interval* | | | | | | | |
